# An Entropy-Based Method with a New Benchmark Dataset for Chinese Textual Affective Structure Analysis

**DOI:** 10.3390/e25050794

**Published:** 2023-05-13

**Authors:** Shufeng Xiong, Xiaobo Fan, Vishwash Batra, Yiming Zeng, Guipei Zhang, Lei Xi, Hebing Liu, Lei Shi

**Affiliations:** 1 College of Information and Management Science, Henan Agricultural University, Zhengzhou 450046, China; 2 School of Computer Science and Mathematics, Keele University, Keele ST5 5AA, UK

**Keywords:** affective structure, corpus annotation, Chinese benchmark datasets, affective computing

## Abstract

Affective understanding of language is an important research focus in artificial intelligence. The large-scale annotated datasets of Chinese textual affective structure (CTAS) are the foundation for subsequent higher-level analysis of documents. However, there are very few published datasets for CTAS. This paper introduces a new benchmark dataset for the task of CTAS to promote development in this research direction. Specifically, our benchmark is a CTAS dataset with the following advantages: (a) it is Weibo-based, which is the most popular Chinese social media platform used by the public to express their opinions; (b) it includes the most comprehensive affective structure labels at present; and (c) we propose a maximum entropy Markov model that incorporates neural network features and experimentally demonstrate that it outperforms the two baseline models.

## 1. Introduction

The development of social media has strongly stimulated the creation of natural language, especially the creation and transmission of subjective emotional expressions in language. Emotional text is predominantly expressed through the thoughts or views of individuals (or groups, organizations, etc.) on characters and events. Chinese emotional text (sentiment) analysis combines linguistic theory and computer technology and uses prior semantic resources to automatically retrieve emotions, attitudes or positions from Chinese texts. However, current approaches are mainly data-driven and heavily rely on large-scale annotated data. Consequently, the emotion description system and annotation resources are the basis for emotion computing.

A good resource for Chinese textual affective structure (CTAS) is the first step toward emotional understanding in artificial intelligence systems. However, the formal description of the CTAS often lacks a reference data set with annotation.

One of the most commonly used representations by researchers is the seven-tuple (originally five-tuple) representation proposed by [1]. Additionally, as emotion is an aspect of semantic expression, frame semantics can be used to describe emotional information [2]. For example,

**Ex.1** 美国人民非常喜欢大熊猫,因为他很像泰迪熊。(English: The American people like the giant panda very much because he resembles a teddy bear very much. )

The structure information can be correctly described by both seven-tuple (“美国人民/holder非常喜期sentiment大熊猫/entity, 因为他很像泰迪熊/reason。”) and frame semantic (“美国人民/experiencer非常/degree喜欢/LU大熊猫/content, 因为他很像泰迪熊/explanation。”) representation. Even though both the seven-tuple and frame semantic representation can correctly describe its structure information, some of them cannot be described in the semantic structure or form of the current framework, such as:

**Ex.2** 这台相机的拍摄效果在夜间不太好。(English: This camera doesn’t shoot very well at night.) We can simply describe it in terms of seven elements of emotion (“这台相机/entity的拍摄效果aspect在夜间/qualifier不太好/sentiment”) but not in terms of frame semantics. On the contrary, the sentence

**Ex.3** 小张在收集火柴盒时获得了极大的满足感。(English: Xiao Zhang finds great satisfaction in collecting matchboxes.) can be represented by frame semantics (小张/experiencer 在收集火柴盒时/content获得了极大的/degree满足。), which is difficult to describe in seven-tuple form.

**Figure 1:**
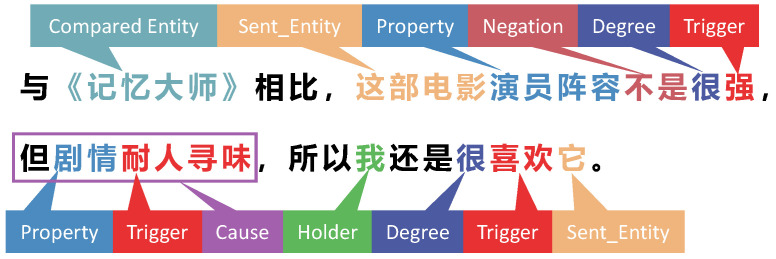
An illustration of a practical annotation case. The English translation of the sentence is “Compared to ’Memento’, this movie doesn’t have a very strong cast. However, the plot is intriguing, so I still enjoyed it”.

To address this problem, our aim is three-fold: to study the description mechanism and resource construction methods of CTAS oriented to information processing, to describe the affective structure comprehensively from the perspective of entity and event logic, and to provide a specially designed model for CTAS task. As shown in Figure 1, we give a practical example of annotation. All of our defined labels appear in this example (the detailed definitions of the labels will be given in Section 3.2). As can be seen, it is easy to determine information about the emotion-holder’s emotional causes, emotion-oriented objects, attributes, and comparison entities after the sentiment structure has been annotated. For example, the overall emotion label of the sentence is ‘*like*’ (the object of its emotion is ‘*this movie*’), which can be inferred due to the trigger word ‘*enjoyed*’, and the cause for ‘*like*’ is ‘*the plot is intriguing*’ (the cause is marked with a box in the illustration because it overlaps with the ‘*Property*’ and the ‘*Trigger*’ labels).

In particular, we present a new dataset that includes 6K short texts labeled for their emotional structure by native speakers. The dataset has a sufficient size and sophisticated annotations. Moreover, unlike many other NLP datasets, the samples of our dataset are taken from Chinese Weibo, where it is more accessible, natural, and easy for native and non-native speakers of Chinese to express their emotions in texts.

From the description mechanism viewpoint, the dataset combines two angles of emotion description methods and presents a unified emotional structure. Firstly, it has a characterization for the entity (and its attributes) and user emotions (views). Secondly, it also reflects the emotion from an event logic perspective, providing all kinds of details. This approach is also beneficial for applications requiring higher-level semantic information.

From the perspective of resource construction, we utilize the existing emotion text databases, frameworks, and semantic resources. We organically merge the entity-level emotion tuples and event-level emotion semantics through artificial alignment and fine-tuning integration to form a unified text emotion resource, which lays the foundation for higher-level tasks like viewpoint detection, position detection and other similar applications.

This dataset will be validated using several machine learning methods commonly used in sequence labeling tasks. Unsurprisingly, conditional random fields (CRF) and encoder representations from transformers with CRF still have excellent performances in our data set. However, our study found that when there are a lot of short comments and the semantic logic of the same sentence is not tightly linked due to the unregulated nature of social media. Based on the reason that CRF is looking for the global final sequence, the above two methods may not be well solved. Therefore, we propose he bidirectional encoder representation from transformers with maximum entropy Markov (BERT + MEMM) model to solve the above problems by using a new feature processing method and MEMM which has better processing effect on local sequences than CRF.

## 2. Related Work

Most of the current corpora are mostly in English, and the Chinese corpus is insufficient, especially the corpus for CTAS, while text sentiment annotation is also a costly task [3]. Xu et al. [4] constructed a sentiment corpus, including elementary school textbooks (Human Education Version), movie scripts, fairy tales, literary journals, etc. In 2020, Chaofa Yuan [5] proposed an annotation scheme based on economic text information, which plays an important role in investment and decision-making. Li Dong [6] introduced artificially annotated Twitter datasets for target-dependent sentiment analysis. His work has beneficially contributed to the development of the field of NLP. Text sentiment analysis is becoming more and more important because the communication of information is greatly enhanced through computer-mediated communication(CMC) [7], such as Weibo, Zhihu, and Tik Tok. In China, Weibo is the most crucial event communication medium, which brings together emotional expressions of the public on various social topics [8]. It is important to study the social sentiment analysis methods for Weibo, and the Weibo text corpus is an important data set for analyzing people’s views on the latest events. Unlike long, standard texts, the Weibo corpus is a relatively informal text with a preference for colloquial speech and short length [9]. Yao et al. [3] applied the corpus to organize the 2nd CCF Conference on Natural Language Processing & Chinese Computing (NLP&CC 2013) Chinese Weibo sentiment analysis evaluation, which strongly promoted the research on Weibo sentiment analysis. However, based on the current performance of sentiment classification of the Weibo text corpus still being unsatisfactory, we, therefore, design a new benchmark dataset to annotate sentences with eight attributes and propose a new method based on Weibo corpus annotation. Sequence labeling task [10], such as Chinese word segmentation [11] and named-entity recognition [12], is a fundamental task in the field of natural language processing [13], in which it needs to assign a label to each element in the sequence. Since the sequence labeling task is called by a large number of subsequent tasks, the performance and efficiency of the sequence labeling model are important. With the support of computing power, coupled with the deep learning algorithm model, sequence labeling is brought to a new stage. In recent years, there have been a large number of pre-training models [14]. It is worth mentioning that pre-training models (such as BERT [15]) have brought significant performance improvement for sequence labeling tasks. The hidden Markov model [16], as one of the earliest models to solve the problem of sequence labeling, has certain limitations in its independent output hypothesis and fusion of complex features.

In 1996, Berger et al. [17] proposed to use the maximum entropy model to solve the problem of part-of-speech (POS) tagging. In 2000, McCallum [18] applied a combination model of the maximum entropy model and the Markov model (MEMM) to solve the information extraction and segmentation tasks. Subsequently, the MEMM is applied in the semantic role labeling [19], human activity recognition using a depth camera [20], Chinese entity extraction [21], and other fields.

## 3. A New Benchmark Dataset

Natural language processing (NLP) systems rely on large-scale training data for supervised training. Currently, most resources are used to mark parts of the information in the emotional structure, such as emotional classification, emotional causes, etc. There are no large-scale resources to mark the whole emotional structure, and most of them are English resources. Influential resources include the film review corpus from Cornell [22], the product review corpus from the University of Illinois at Chicago (UIC) [23], the MPQA corpus [24], a restaurant review corpus from MIT, and a Chinese hotel review corpus from Dr. Tan Songbo of the Chinese Academy of Sciences. The most prominent problem in the field of Chinese NLP is the lack of data, especially the lack of large-scale annotation resources of CTAS based on appropriate description mechanism, emotion classification, emotion reasoning, and the whole emotion structure. This study was carried out according to the annotation tool, corpus selection, and corpus annotation. Six thousand subjective Chinese texts were labeled with emotional structure information, and the description of emotional information was studied from both the perspective of the entity and the event.

### 3.1. Affective Structure Annotation Tool

Fully manual annotation (independent of the annotation system) is time-consuming. Therefore, it is important to choose open-source tools that are lightweight and efficient with text-boundary (SPAN) annotations. To address the challenges above, after many comparisons, we chose YEDDA, a lightweight and efficient annotation tool for text span annotation [25]. The comparison of annotation tools is shown in Table 1. YEDDA (the previous SUTDAnnotator) is developed for annotating chunk/entity/event on text (almost all languages including English and Chinese), symbol and even emoji. It supports shortcut annotation, which is highly efficient in annotating text by hand. The user only needs to select text span and press the shortcut key, and the span will be annotated automatically. It also supports the command annotation model that annotates multiple entities in batch and supports exporting annotated text as a text sequence. In addition, a concise and crisp user interface page is also very friendly for native Chinese annotators.

### 3.2. Corpus Selection

**Figure 2:**
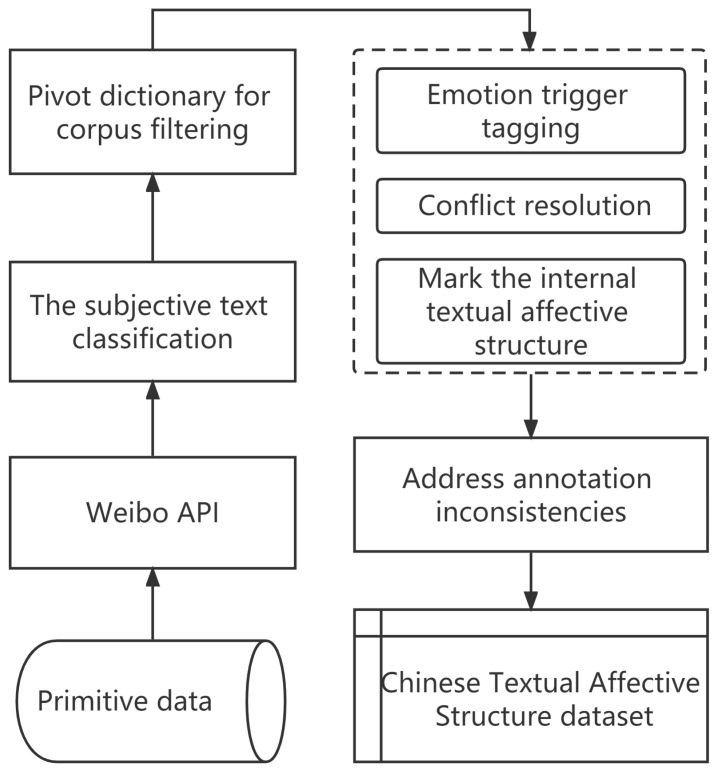
Annotation Framework.

**Figure 3:**
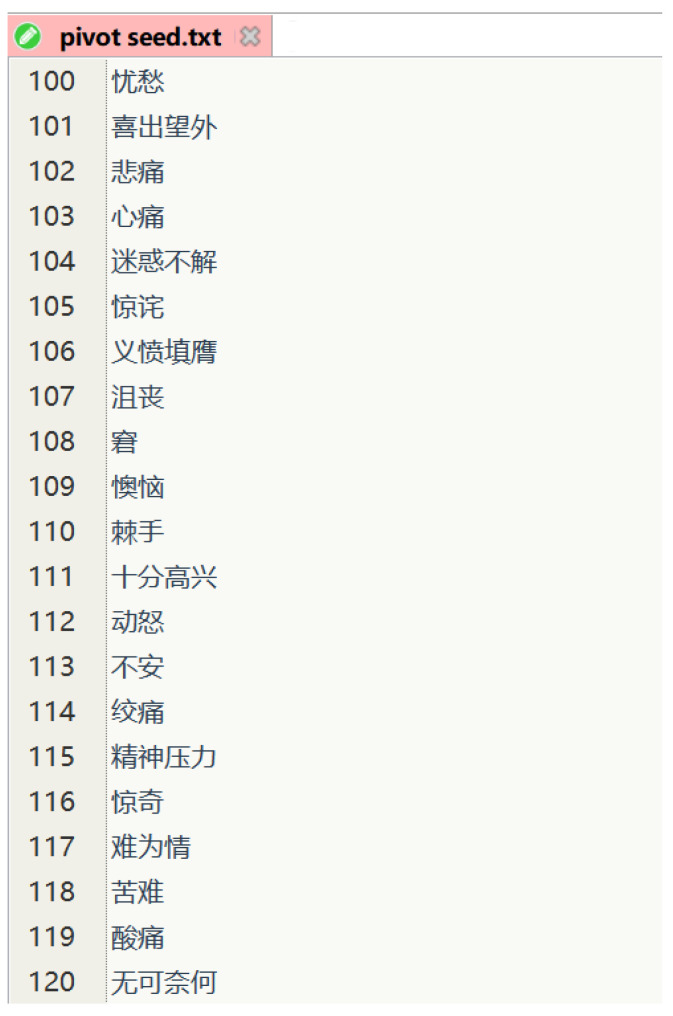
A pivot seed fragment. The English translations of the Chinese seed words in the examples are “sad, overjoyed, sad, heartbroken, confused, surprised, indignant, frustrated, embarrassed, regretful, troublesome, very happy, angry, uneasy, colic, mental stress, surprised, embarrassed, suffering, sore and helpless”.

Training current artificial intelligence models requires the support of a massive corpus [26], so several types of textual corpus sources have been formed. The first type is news texts of official media [27], and the second type is Internet-user-generated texts [28]. The second type can be divided into long texts and short texts. Emotion-expressing texts are mainly concentrated on personal web-media platforms, such as Weibo. Therefore, the source of our corpus is determined to be the Weibo platform. We chose simplifyweibo_4_moods (https://github.com/SophonPlus/ChineseNlpCorpus, accessed on 2 February 2019) as the base corpus, which is commonly used for emotion classification tasks [29,30,31,32]. The overall pre-processing process is as follows. The first step is to collect the raw data and de-duplicate it using the Weibo Application Programming Interface. The second step is to manually label the Weibo content with emotion label. The third step is content filtering based on the pivot trigger lexicon. The final step is the manual annotation of the corpus. Our overall labeling process is shown in Figure 2. And, a typical pivot fragment is shown in Figure 3.

### 3.3. Affective Structure Labeling

Through the adjustment and fusion of text emotion tuple and framework semantic (emotion category) representation, we propose a new unified expression form of Chinese text emotion structure from another level to describe Chinese text emotion structure more precisely. Our Chinese label text emotional structure has the following design principles: (a) determine the emotional trigger word template, types, and annotation type composition; (b) perform conflict resolution between principles and resolution mechanisms; (c) determine the operability and consistency of the process; and (d) determine the emotional information description from both entity and event perspectives. The label design and interpretation are shown in Table 2.

Due to the purpose of this study, building an emotional structure can be used as a gold standard Chinese annotation resource. Therefore, the artificial tagging corpus research method was used, first, by machine algorithm segmentation mark, and after, letting Chinese native speakers’ personal tagging finally make traditional domain experts examine personal marks from inconsistent decisions. Although the Weibo corpus is full of irregularities and uncertainties, in the annotation process, unified annotation standards must be observed so as to decrease migration, speed up annotation, reduce the difference caused by different annotation personnel, and achieve improved annotation consistency. Here are some annotation specifications and examples.

**1.**  **The entity labeling range should be as long as possible rather than as short as possible.**
  **Example:** 天有不测风云啊！广东暴雨呀！落雨大，水浸街。看看这些可怜的轿车。(English: Things can happen! What a terrible Guangdong rainstorm! It rained heavily and flooded the street. Look at these poor cars.)
  **Judgement:** This short comment means to transmit the pitiful pity for the car. The direct reason is that “it is raining heavily, flooding the street”, but the front of the “weather is unpredictable! ”Guangdong rainstorm” also belongs to the cause of emotion. So this short comment should be “the day has an unexpected storm! Torrential rain in Guangdong! It’s raining hard and flooding the streets. ” Labeled “cause”.

**2.**  **It is necessary to judge whether some words commonly used to express emotions according to the actual situation.**
  **Example:** 超级喜欢啊啊啊阿啊啊啊啊啊啊, 樱桃小丸子，超可爱∼∼ 樱桃小丸子同学很小很小的时候。你喜欢吗？ (English: I really like it ah ah ah ah ah, Sakura Maruko, super cute ∼∼ when Sakura Maruko was very young. Do you like it?)
  **Judgement:** “Like” is a common expression of emotion, but it does not work in every situation. This short commentary can be judged from the context to express the lovely love for Chibi Maruko-chan. Therefore, the first “like” needs to be marked trigger to express liking, but the second “like” does not need to be marked

**3.**  **Negation cannot appear alone, that is, in the absence of the trigger, and even if there are negation words, they cannot be marked.**
  **Example:** 最近的伙食不太满意，都是吃外卖，一点也不喜欢。 (English: I’m not very satisfied with the food recently; they all eat takeout. I don’t like it at all.)
  **Judgement:** From the brief comments, it can be judged that Holder wants to express the mood is not happy. Therefore, the first “no” is not a modification of emotion and does not need to be marked negation. The second “no” expresses Holder’s unhappiness and needs to be marked.

**4.**  **Annotate the main emotional expression subjects of brief comments. That is, when one emotional expression is a part of another emotional expression, we find the main emotional expression to mark, and the other emotional expressions as the cause or other entity.**
  **Example:** 新人真的好难带啊，我要疯特了。虽说我也是这么过来的，但是这样新的还是突破我笨的极限了。啊，神呐，救救我吧。(English: It’s really hard to bring a newbie, I’m going crazy. Although I also have the same experience, this new one still breaks through the limit of my stupidity. Oh my God, please save me.)
  **Judgement:** According to the short comments, it is clear that Holder is mad and helpless. “I’m going crazy. ” “Oh my god, please save me.” Higher levels of emotional expression. Holder’s madness should be included in this sentiment.

**5.**  **Ensure that each sentence has an emotional trigger; that is, if there is no obvious emotional trigger, the weak emotional trigger should also be marked.**
  **Example:** 每天都希望期待更多的人粉我我也希望粉大家。加油-微笑天使互粉群。 (English: I hope to expect more people to follow me every day, and I also hope to follow everyone. Come on, smile Angel Mutual Fan Group.)
  **Judgement:** There is no obvious emotional trigger in the brief review. We judge that the brief comment expresses the weak emotion “hope” according to the context, so we need to mark “hope” as a trigger.

**6.**  **In the absence of obvious emotional words, some blessing phrases can usually express the core emotion of the whole sentence and can be used as emotional trigger words.**
  **Example:** 祝大家中秋快乐！自制板栗月饼：自己动手，丰衣足食。今年中秋也不例外吧。月饼的详细做法：更多中秋菜谱。 (English: Happy Mid-Autumn Festival to everyone! Homemade chestnut Mooncakes: Do it Yyurself, get well-dressed. This year’s Mid-Autumn Festival is no exception. Details of Mooncakes: more Mid-Autumn recipes.)
  **Judgement:** There is no obvious trigger in this brief comment., but it is not hard to see that the whole paragraph is intended to express the joy of the Mid-Autumn Festival, so you can put “Happy Mid-Autumn Festival to everyone!” as a trigger.

Table 3 shows the general statistics of CTAS. The number of microblogs obtained by filtering and then tagging is 6806, and the number of labels in each category is detailed in the table. The data is stored in CoNLL format, i.e., the first column is the token, the second column is the corresponding BIO tags, and we also have an additional third column of POS tagging features provided. As benchmark data for machine learning models, the dataset is split using stratified sampling (ratio 8:1:1) to obtain training, validation, and testing sets. The benchmark is available at https://github.com/pdsxsf/CTAS (accessed on 1 January 2022).

## 4. Dataset Benchmarking

To see how the nine-element description can be used in the formal description and resource construction of CTAS detection, we have used several of the most popular methods to benchmark datasets, especially the BERT-based maximum entropy hidden Markov model (BERT + MEMM).

### 4.1. Methodology

**Figure 4:**
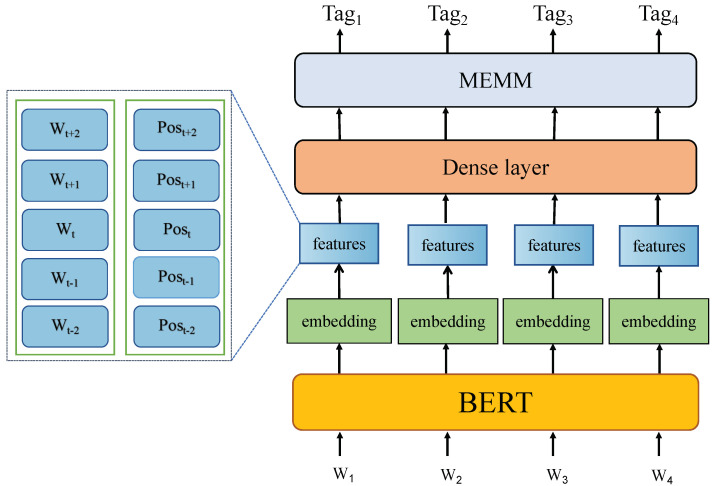
The framework of our model.

In this section, we propose the bidirectional encoder representation from transformers+maximum entropy Markov models (BERT + MEMM) to solve the limitations of CRF in calculating the globally optimal output node. Our approach is divided into two parts: feature building and maximum entropy Markov models. Figure 4 illustrates our model structure. First of all, in the feature construction part of the model, BERT, which is popular in sentiment analysis tasks, is used as the pre-training model of the model and combined with the traditional N-gram feature construction. The new feature vector formed by the integration of tri-gram and embedding of the text output by BERT will be input into the fully connected neural network for feature transformation. Then, the ground truth will be used to guide the training process to maximize the probability of correct label. Finally, MEMM will be used for optimal sequence decoding in the test process.

#### 4.1.1. Model Feature Processing

BERT’s main model structure is a transformer encoder, which has a wide range of applications in sentiment analysis tasks. The BERT model uses two pre-training objectives to complete the learning of text content features. One is the masked language model (MLM), which is used to predict masked words by covering words and learning contextual content features. The other is masked next sentence predication (NSP), which is masked by learning relationship features between sentences and predicts whether two sentences are next to each other. Because maximum entropy Markov models, namely max-entropy Markov models, introduce a first-order Markov hypothesis on the basis of maximum entropy, the current state is only related to the previous state. Suppose we have a sentence of n words S=(w1,w2,⋯,wn). Each word wi depends on the effect from the first word w1 to the word wi−1 before it.

(1) $$p\left(S\right)=p\left(w_{1}w_{2}\cdots w_{n}\right)=p\left(w_{1}\right)p\left(w_{2}|w_{1}\right)\cdots p\left(w_{n}|w_{n-1}\cdots w_{2}w_{1}\right)$$

This hypothesis can effectively solve the problem of the large parameter space of the N-gram language model, which is widely used in sequence-labeling feature construction. The spatial complexity of the N-gram model is an exponential function of N:

(2) $$O\left({\left|V\right|}^{N-1}\right)$$

Here, |V| refers to the number of words in a language dictionary. Experience shows that the effectiveness of language modeling is greatly enhanced when the value of N is set to 3, compared to a value of 2. Increasing the value to 4 gives an improvement over the tri-gram, but the computational resource consumption increases even more. So we use tri-gram to solve the long-range dependency problem of the model [33]. We concatenated the embedding of the BERT output with the tri-gram vector to form a 4101-dimensional vector to be used as text features. The fused feature vectors were then fed into a fully connected neural network for feature transformation.

#### 4.1.2. MEMM

MEMM is an entropy-based model. Entropy is a measure of the uncertainty of a random variable. The greater the uncertainty, the greater the entropy. Suppose the probability distribution of discrete random variable X is P(x), then its entropy can be calculated by the equation:

(3) $$H\left(P\right)=-\sum_{x}P\left(x\right)\log P\left(x\right)$$

There are two principles for learning probabilistic models in the context of maximum entropy: the first one is to acknowledge what is known (knowledge). The second one is to make no assumptions about the unknown, without any bias, but also to ensure that the entropy is maximum, not taking sides in the unknown. A simple example of a part-of-speech (POS) tagging task is that the word “learn” can be labeled as either a $y_{1}$ noun or a $y_{2}$ verb. At the same time, we provide the parts of speech of the preceding word as $x_{1}$ noun, $x_{2}$ adjective, $x_{3}$ verb, $x_{4}$ adverb. In the absence of any limitation, the principle of maximum entropy holds that any explanation is equally probable. In other words,

(4) $$P\left(y1|xj\right)=P\left(y1|xj\right)=\frac{1}{8},1<j<4$$

There are usually many constraints in the actual task, such as the adverb at the beginning, and the probability of learning as a verb becomes very large. Therefore, we design a characteristic function f(x,y) to describe a certain fact between input x and output y, which has only two values of 0 and 1, which is called a binary function. That is,

(5) $$f\left(x,y\right)=\left\{\begin{array}{c} 1,\;\mathrm{if}\;y\;\mathrm{is}\;\mathrm{the}\;“\mathrm{Noun}”\;\&\;x\;\mathrm{is}\;\mathrm{the}\;“\mathrm{Adverb}”\;\&\;x\;\mathrm{is}\;\mathrm{the}\;\mathrm{beginning}\\ 0, \end{array}\right.$$

The maximum entropy principle selects the optimal probability distribution under characteristic constraints similar to those above. The fact that the maximum entropy has no independent assumptions on the selected features can solve the problem that most prediction models, such as decision tree, logistic regression, and neural network, make some wrong assumptions about regarding information. In addition, any complex features can be used, but the maximum entropy model does not consider the time sequence relationship. With the observation sequence as the condition, each word is judged separately, and the relationship between states cannot be fully utilized. The hidden Markov model (HMM) considers the observation as an output value related to the current state at the moment. The HMM with temporal relationship can establish the Markov property between the states, which can make up for the problem that the relationship between the states of the maximum entropy model cannot be fully utilized. So, it was a natural idea to combine the advantages of the two, which led to the MEMM. The idea of the MEMM is to use the HMM framework to predict the sequence labels of a given input sequence while combining it with a maximum entropy approach to obtain greater freedom in the type and number of features extracted from the input sequence.

When we deal with sequence labeling, the problem we need to solve is to predict the POS or entity for a sentence sequence. In fact, we need to predict the POS or entity sequence $\left(s_{1},s_{2},s_{3},s_{4}\right)$ and sentence sequence $\left(o_{1},o_{2},o_{3},o_{4}\right)$, it is more reasonable to take the information (feature) contained in the text context as the judgment basis of decoding output state in the calculation process. The hidden Markov model can help us solve such a problem. For example, given the model λ and a particular observation (output) sequence, the HMM finds the hidden state sequence that is most likely to produce this output sequence. Specifically, with all possible hidden state sequences listed, the HMM calculates the model that maximizes the joint probability of the observed sequence and the hidden state sequence using the following equation:

(6) $${\mathrm{argmax}}_{s}P\left(s_{1},s_{2},s_{3}\cdots ,o_{1},\;o_{2},\;o_{3}\cdots |\lambda \right)=\prod P\left(s_{t}|s_{t-1}\right)*P\left(o_{t}|s_{t}\right)$$

### 4.2. Baselines

In order to evaluate the effectiveness of our annotated data and the proposed model in the field of Chinese textual affective structure analysis, we used two classes of classical models with the proposed approach on our annotated dataset for comparison.

**CRF** The conditional random fields (CRF) model is the worst performer, as expected. The CRF algorithm involves two kinds of characteristic functions; one is the state characteristic function, which calculates the state score, and the other is the transfer characteristic function, which calculates the transfer score. The former only focuses on what entity tags the characters at the current position can be converted into, while the latter focuses on what combination of entity tags the characters at the current position and its adjacent positions can have. In the case of CRF only, the above two types of eigenfunction are set manually. Popularly speaking, it is the feature that sets the observation sequence manually, as in, for example, artificial state feature templates, such as “a word is a noun” and artificial transfer feature templates, such as “when a word is a noun, the last word is an adjective”, etc. The performance of entity recognition depends on how well the two feature templates are set.

**BERT + CRF** In order to solve the excessive dependence of CRF model on artificial feature construction and reduce errors in sequence labeling, we adopted bidirecctional encoder representation from transformers + the conditional random fields, which is widely used in the sequence annotation tasks model (BERT + CRF) method. BERT learns the state features of the sequence and obtains a state score, which is directly input to the CRF layer without manually setting the state feature template. Here, state refers to the state sequence that may correspond to a particular location (named-entity recognition refers to the entity annotation), state grading refers to the state before every possible softmax risk (also called informal, or directly called the score) and is usually stressed in the BIO entity tagging “B began to say the word, and I said the last word, which is the real word O.” For example, the following sentence and the corresponding entity label (assuming the person and the place we want to identify): “小明爱北京的天安门。 (English: Xiao Ming loves Tiananmen in Beijing.)” “B-person O b-location i-location O b-location i-location i-location O”. The entity labeling corresponding to the output maximum score may still be wrong, and it will not be 100% correct. It is possible for B to be directly followed by B, and the latter labeling starts with I. Using CRF can reduce the occurrence probability of these obvious irregularities and further improve the accuracy of BERT model prediction. However, since CRF computes the conditional probability of the globally optimal output node, it has certain limitations for tweets that are not restricted by any rule logic. There are a large number of short and non-standard comments and semantic mutation of the same comment in the microblog book, which cannot be effectively solved by CRF. Additionally, BERT + CRF training is costly and complicated. Therefore, BERT + MEMM is proposed to solve this problem.

## 5. Experiments and Result Analysis

### 5.1. Experimental Setup

This section includes lab settings, evaluation indicators, and our findings. Section 5.1 describes the process details and parameters of the experiment, Section 5.2 describes the performance assessment measures to assess the performance of the proposed method, and Section 5.3 describes the analysis of the results obtained to determine the effectiveness of CTAS in the mechanism and construction of emotional description of Chinese text. Based on CTAS as benchmark dataset, we use bi-gram and POS information as feature input on the CRF model. In general, performance of a neural networks is inspired by hyperparameters such as the number of hidden neurons in the hidden layer or values of decay variable which restricts neuronal connections weight. In this study, BERT took the pre-trained model from huggingface and fine-tuned it. All models are tuned with data sets. Table 4 provides detailed settings for the baselines. The accuracy and weighted F1 score were used as performance indicators. All models are tuned with data sets. Table 4 provides detailed settings for the model. The accuracy and weighted F1 score were used as performance indicators.

### 5.2. Evaluation Metrics

The text data of microblog comments are imbalanced. In order to improve the robustness of the model, precision, recall and F1 scores based on confusion matrix are used to measure the model performance. These measurements are calculated with the following equations:

(7) $$\;\mathrm{Precision}\;=\frac{TP}{\left(TP+FP\right)}$$

(8) $$\;\mathrm{Recall}\;=\frac{TP}{\left(TP+FN\right)}$$

(9) $$F1=\frac{2\times \;\mathrm{Precision}\;\times \;\mathrm{Recall}\;}{\left(\mathrm{Precision}\;+\;\mathrm{Recall}\right)}$$

*TP*, *FN*, and other symbols used in the formula are explained as follows:

True Positive (*TP*): indicates the number of true positive samples. The real category of samples is positive, and so is the result of model recognition.

False Negative (*FN*): indicates the number of false negative samples. The true category of a sample is a positive category, but the model recognizes it as a negative category.

False Positive (*FP*): indicates the number of false positive samples. The true category of the sample is a negative class, but the model recognizes it as a positive class.

True Negative (*TN*): indicates the number of true negative samples. The true category of the sample is a negative class, and the model recognizes it as such.

### 5.3. Result Analysis

We conducted a series of experiments to measure the performance of the proposed CTAS in terms of the Chinese emotion description mechanism. The experimental results are shown in Table 5. In our test set, BERT + MEMM achieved 61.22%, 54.85%, and 56.13% respectively in precision, recall, and F1 scores. In terms of F1 values, our model achieved 56.13%, much higher than that of other partners. Our model performed better on both the precision and recall metrics, while the shortcomings of CRF and BERT + CRF on their respective precision and recall metrics resulted in lower combined F1 scores. That is to say, for tweets with a large number of short comments and possibly weak logic for the next sentence, MEMM correctly identifies the boundaries of the elements without loss of accuracy.

In terms of precision, the accuracy of CRF was 62.84%, much higher than that of BERT + CRF (53.9%), while our BERT + MEMM achieved a similar score (61.22%). Precision represents the proportion of tags that the model predicts to be positive to be correct. From our experimental results, it is clear that in this task, the distributed features of the BERT output combined directly with the CRF will instead reduce the precision.

In terms of recall, the score of BERT + CRF (55.64%) was higher than that of CRF (43.63%) and BERT + MEMM (54.85%). Recall refers to the proportion of tags that are predicted correctly by the model from all positive tags. This experimental result also shows that the feature fusion ability of BERT is relatively strong, which can make better use of the context information provided by the text to be recognized.

**Figure 5:**
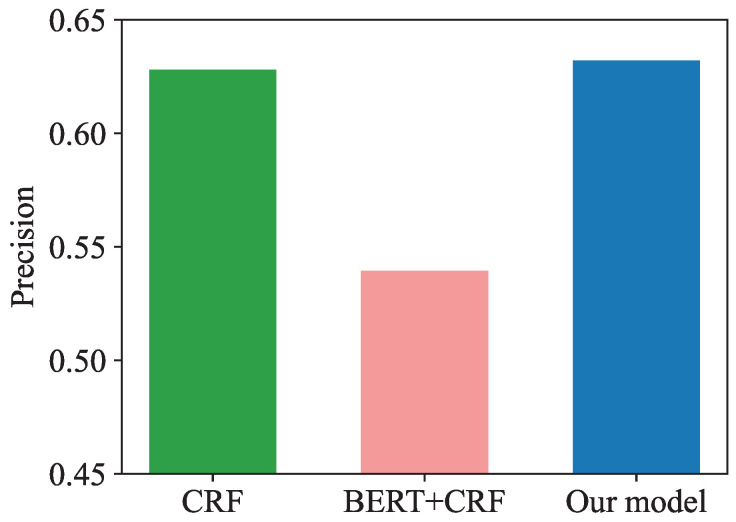
Model precision comparison.

**Figure 6:**
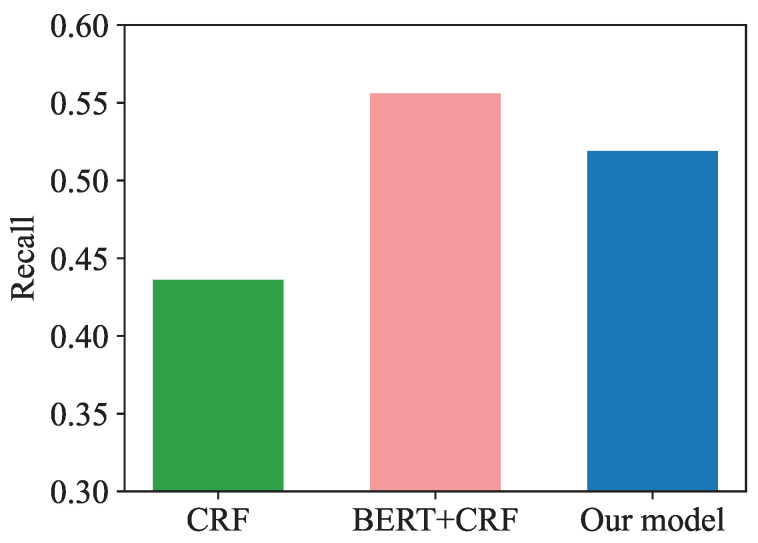
Model recall comparison.

**Figure 7:**
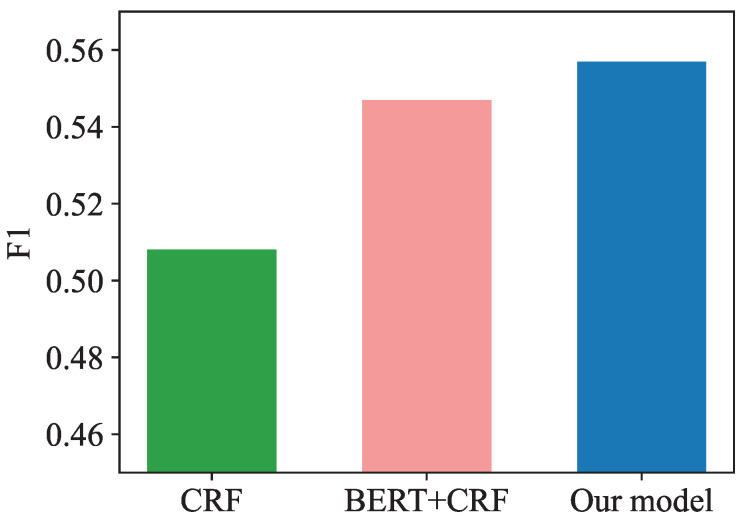
Model F1-score comparison.

In order to provide a more visual representation of the model performance comparison, we have drawn bar comparison charts, as shown in Figure 5, Figure 6 and Figure 7.

## 6. Conclusions

In this work, we introduce the CTAS benchmark data, a new dataset for the mechanism of Chinese emotion description detection for machine learning. Our work provides a reasonably large dataset which can be helpful for the development of artificial intelligence’s understanding of emotion in the field of natural language processing. Our raw data are collected from Weibo posts, where it is possible to investigate emotional expression mechanisms for Chinese text. We show that identifying the Chinese emotional expression mechanism is difficult in sequence annotation and automatic classification. At the same time, it also forms a unified text emotion annotation resource, which lays a data foundation for the application of higher-level viewpoint discovery and position detection. In the text, we propose the BERT + MEMM method to better solve the sequence labeling problem for Chinese microblog text. As the first Chinese emotional expression benchmark dataset, it provides opportunities for the development of the Chinese textual affective emotion field.

## Figures and Tables

**Table 1:** Annotation Tool Comparison.

Tool		WordFreak	GATE	Stanford	Atomic	Anafora	BRAT	YEDDA
Operating System	MacOS	Yes	Yes	Yes	Yes	Yes	Yes	Yes
	Linux	Yes	Yes	Yes	Yes	Yes	Yes	Yes
	Win	Yes	Yes	Yes	Yes	No	No	Yes
Self-Consistency		No	Yes	Yes	Yes	No	No	Yes
Command Line Annotation		No	No	No	No	No	No	Yes
System Recommendation		Yes	Yes	No	No	No	Yes	Yes
Analysis		No	No	No	No	No	No	Yes
Size		1.1 M	544 M	88 k	5.8 M	1.7 M	31.7 M	80 k
Language		Java	Java	Java	Java	Python	Python	Python

**Table 2:** Description of labels in the labeling system.

Label	Paraphrase
Cause	The thing that makes emotions happen.
Degree	The level or amount of emotions.
Holder	The person or people who hold the emotions.
Negation	To express the emotions are not exist.
Property	A characteristic that entity has.
Trigger	The expression of emotions.
Compared_Entity	The entity be compared or related to emotions.
Sent_Entity	The entity be sent with emotions or receive emotions.

**Table 3:** Statistics of CTAS Dataset.

	Full Set	Train	Val	Test
# of Microblog	6806	5443	681	682
# of Trigger	9424	7574	897	953
# of Sent_Entity	3534	2824	354	356
# of Property	255	205	26	24
# of Negation	407	318	30	59
# of Holder	2247	1820	212	215
# of Degree	5268	4241	502	525
# of Compared_Entity	73	62	4	7
# of Cause	1123	898	120	105

**Table 4:** Parameter Settings.

	CRF	BERT + CRF	BERT + MEMM
**Input Features**	**Unigram, Bigram, POS**	**Word Sequence**	**Word Sequence, Tigram**
Sequence length	-	64	64
Batch size	-	128	128
Learning rate	-	3 $\times \;10^{-4}$	3 $\times \;10^{-4}$
Training epochs	-	10	5

**Table 5:** Comparison of precision, recall, and F1 values of different methods.

	Precision	Recall	F1
CRF	0.6284	0.4363	0.5085
BERT + CRF	0.5395	0.5564	0.5479
BERT + MEMM (Our model)	0.6122	0.5485	0.5613

## Data Availability

The benchmark is available at https://github.com/pdsxsf/CTAS (accessed on 1 January 2022).
